# Onlay Versus Sublay Mesh Repair for Incisional Hernias: A Systematic Review

**DOI:** 10.7759/cureus.34156

**Published:** 2023-01-24

**Authors:** Chirag Pereira, Shankar Gururaj

**Affiliations:** 1 General Surgery, Royal Lancaster Infirmary, Lancaster, GBR; 2 General Surgery, Father Muller Medical College and Hospital, Mangalore, IND

**Keywords:** incisional hernia, mesh repair, hernia, sublay, onlay

## Abstract

Incisional hernias are a common problem following major abdominal surgery. There are numerous surgical techniques described in the existing English scientific literature with different planes for mesh placement. The current review aims to compare onlay versus sublay repair in managing incisional hernias. A systematic literature search was conducted on Embase, the Cochrane Library, PubMed, and Medline to identify randomised controlled trials (RCTs) comparing onlay versus sublay mesh repair for incisional hernias. We identified six RCTs that included 986 patients, of whom 503 were in the onlay group and 485 were in the sublay group. There was no statistically significant difference in hernia recurrence between the onlay and sublay groups (odds ratio (OR): 1.3 (0.49-3.47), 95% confidence interval (CI), p=0.60). Seroma formation was significantly higher in the onlay group (OR: 2.85 (1.74-4.67), 95% CI, p<0.0001). There were 45 reported cases of surgical site infection (SSI). There was no significant difference between the two groups (OR: 1.46 (0.44-4.84), 95% CI, p=0.54). Haematomas were reported in 11 cases, and there was no significant difference between the two groups (OR: 2.13 (0.56-8.19), 95% CI, p=0.27). Four RCTs reported the length of the hospital stay. There was no significant difference between the two groups (mean difference (MD): 0.53 (-0.16-1.22), 95% CI, p=0.13). We failed to draw conclusive clinical recommendations due to the variability in the included RCTs. We recommend well-structured, large-volume RCTs to better compare these two surgical techniques.

## Introduction and background

Incisional hernia (IH) is a common complication following major abdominal surgery such as laparotomies, with an incidence ranging between 2% and 20% [1]. The aetiology is multifactorial and often divided into patient-related factors and technical failure. Common patient-related factors include morbid obesity, malnutrition, anaemia, diabetes, and immunocompromised status, while technical failure can occur depending on the type of suture material used, the length of the suture material in relation to the length of the incision, and the type of fascial closure [2].

The two surgical approaches for the management of IH are open surgery and minimally invasive surgery. Both techniques make use of a mesh to reinforce the repaired defect by means of a fibrotic reaction in tissue and reduce the chance of hernia recurrence [3]. The ideal plane for mesh placement is debatable, but the most common anatomical locations include sublay, inlay, and onlay. In sublay, the mesh is placed behind the rectus muscle and in front of the posterior rectus sheath, whereas in the inlay technique, the mesh is anchored in line with the fascial defect. Onlay mesh is placed directly on top of the anterior fascia [4].

A systematic review conducted in 2014 compared onlay with sublay mesh repair but included only two randomised controlled trials (RCTs) at that time [5]. Our study aims to review newer published RCTs comparing onlay with sublay mesh repair for open incisional hernias. The outcomes evaluated were postoperative complications, recurrence of the hernia, and length of hospital stay.

## Review

This scientific study was planned and conducted according to Preferred Reporting Items for Systemic Reviews and Meta-Analyses (PRISMA) [6].

Search strategy

A detailed literature search of PubMed, Medline, the Cochrane Library, and Embase was performed independently by two authors. The search was limited to human studies with no language or publication date restrictions. Boolean operators AND and OR were used along with the following keywords: "incisional hernia" AND "mesh" OR "onlay" OR "sublay" OR "retrorectus" OR "subfascial" OR "postoperative complications".

**Figure 1:**
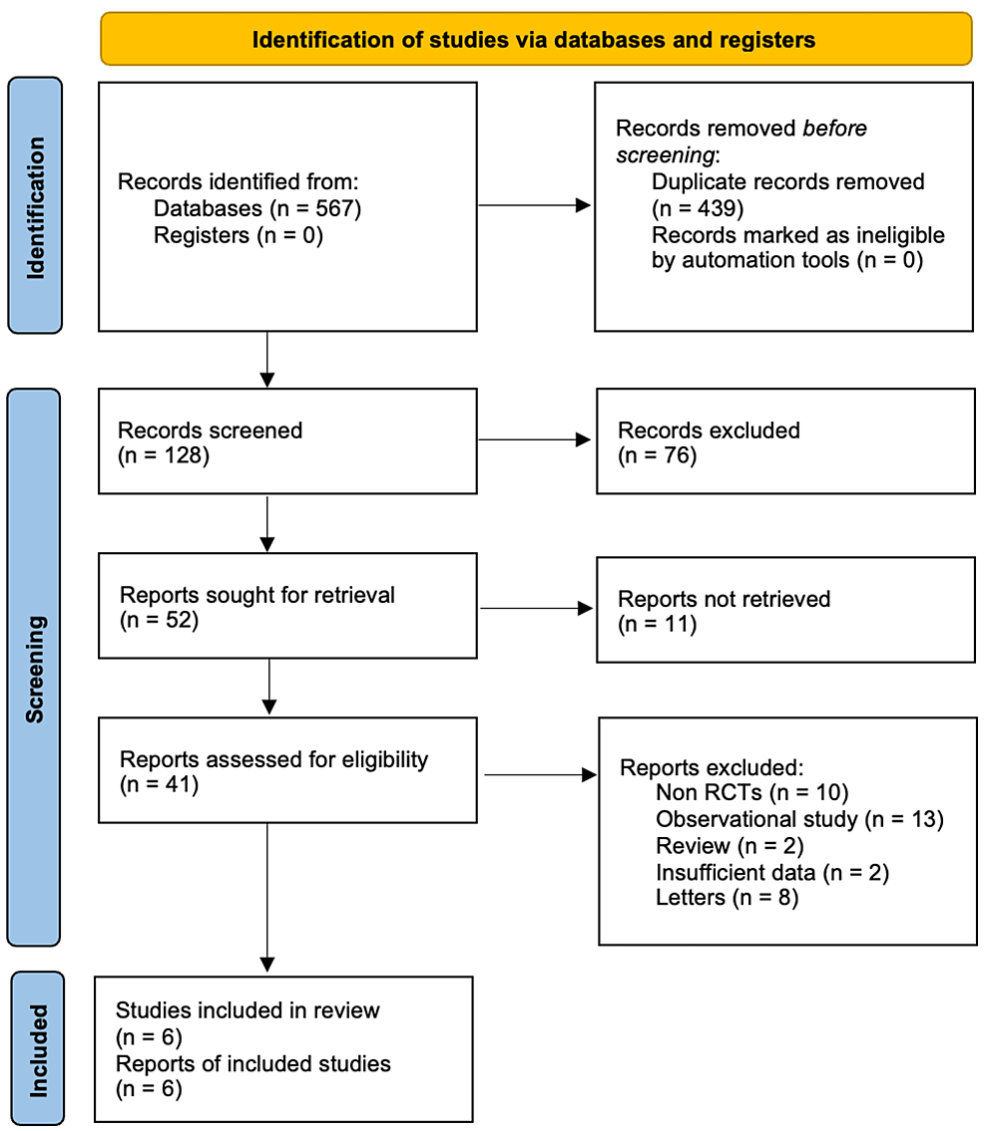
PRISMA flow chart PRISMA: Preferred Reporting Items for Systemic Reviews and Meta-Analyses The electronic search retrieved 567 records. After the removal of duplicates, we had 128 studies. These studies were screened by title and abstract. Screening retrieved 52 studies, of which 11 reports could not be retrieved, and hence 41 reports were available after screening. 35 studies were excluded as they did not meet our inclusion criteria, leaving six studies for analysis.

Studies were included if they met the following criteria: RCTs comparing onlay versus sublay repair for incisional hernias, non-RCTs, case reports, observational studies, reviews, and studies where additional procedures were carried out in relation to incisional hernia repair were excluded. Studies comparing ventral hernias, such as umbilical and paraumbilical hernias, were not included. After the database search, 567 records were identified. After the removal of duplicates, 128 studies were screened. 76 studies were excluded based on the review of the abstract and title (Figure 1).

Data collection

The following data were collected: first author and year of publication, study country of origin, population distribution, gender distribution, mean age, post-op complications, duration of follow-up, hospital stay, and recurrence. Data were collected by two authors separately and inputted into a Microsoft Excel sheet (Microsoft Corporation) to ensure conformity.

Assessment of risk of bias

**Figure 2:**
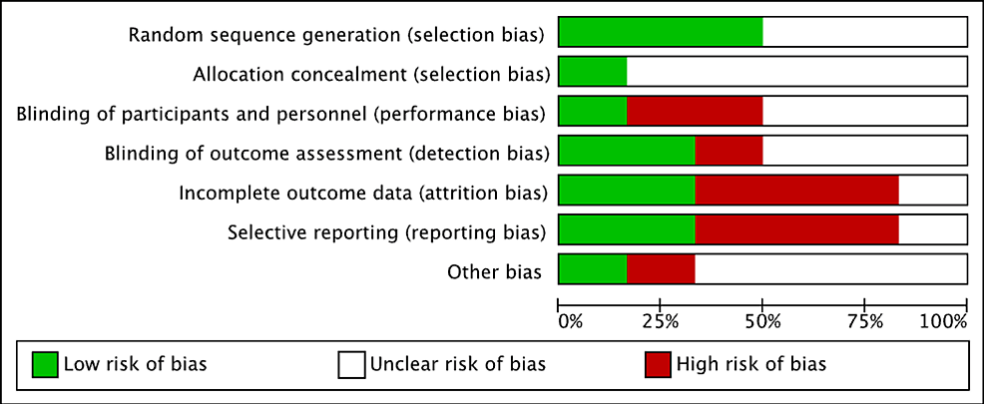
Risk of bias assessment

The risk of bias was assessed based on the Cochrane risk-of-bias tool [7] by two authors. The following categories were classified as low, high, or unclear: random sequence generation, allocation concealment, blinding of outcome assessment, blinding of participants and personnel, selective reporting, and other sources of bias. There was an unclear method of randomization in three studies. The risk of selection bias was low. Some studies had incomplete data outcomes and reporting, which resulted in a higher risk of reporting and attrition bias. Discrepancies in the interpretation of the risk of bias were resolved by mutual agreement between authors (Figure 2).

 

Statistical analysis

Review Manager 5.4 was used for statistical analysis. The odds ratio at a 95% confidence interval was calculated for dichotomous variables. Random effect models and fixed effect models were used for analysis appropriately. The Cochran's Q test and the I2 test were used to assess heterogeneity in the included studies. 0% was considered no heterogeneity, while >50% was considered significant heterogeneity. p-value was assessed using a forest plot. A value of <0.05 was considered to be statistically significant.

Results

Six RCTs were included in our analysis, comprising a total of 986 patients. There were 503 patients in the onlay group and 483 patients in the sublay group. Characteristics of the included studies and population characteristics are shown in Table 1 and Table 2, respectively.

**Table 1:** Characteristics of included studies BMI: body mass index; ASA: American Society of Anesthesiologists Table 1 shows the number of included studies in the systematic review along with the description of the included population and the follow-up period.

Author	Country (year of publication)	Population distribution (onlay / sublay)	Description of population	Length of follow-up (months)
Venclauskas et al. [8]	Lithuania (2010)	57 / 50	Not recorded	12
Weber et al. [9]	Hungary (2010)	235 / 224	Inclusion criteria: ages 18-70, abdominal wall hernias; exclusion criteria: a systemic disease that usually adversely affects wound healing; other surgeries performed at the same time	60
Demetrashvili et al. [10]	Georgia (2017)	78 / 77	Inclusion criteria: midline primary incisional hernia; exclusion criteria: recurrent incisional hernia, strangulated hernia	Not recorded
Sevinc et al. [11]	Turkey (2018)	50 / 50	Inclusion criteria: midline incisional hernias; exclusion criteria: BMI of above 40 kg/m^2^, ASA 4, or severe pulmonary or cardiac disease	36
Ahmed et al. [12]	Pakistan (2019)	33 / 32	Inclusion criteria: symptomatic incisional hernia; exclusion criteria: previous history of chronic illness like diabetes mellitus, use of steroids, and presentation with signs and symptoms of obstruction and strangulation	6
Kumar et al. [13]	India (2021)	50 / 50	Inclusion criteria: ages 18-70 years; symptomatic incisional hernias; exclusion criteria: <18 years of age; transverse incisional hernias	Not recorded

**Table 2:** Characteristics of the study population BMI: body mass index; SD: standard deviation Table 2 compares the onlay population group with the sublay population group with respect to age, sex, and BMI.

Study	Onlay group			Sublay group		
	Age (years) Mean ± SD	Male/ Female	BMI (kg/m^2^) Mean ± SD	Age (years) Mean ± SD	Male/ Female	BMI (kg/m^2^) Mean ± SD
Venclauskas et al. [8]	56.9 ± 11.5	22/35	30.5 ± 7.4	53 ± 11.6	23/27	28 ± 6
Demetrashvili et al. [10]	61.2 ± 16.7	32/46	28.8 ± 3.9	59.6 ± 13.1	36/41	29.4 ± 4.7
Sevinc et al. [11]	55.9 ± 11.8	22/28	25.5 ± 3.5	55.9 ± 12.1	14/28	26.4 ± 3.3
Ahmed et al. [12]	Not recorded	20/13	Not recorded	Not recorded	22/10	Not recorded
Kumar et al. [13]	Not recorded	32/18	Not recorded	Not recorded	29/21	Not recorded

Hernia recurrence

**Figure 3:**
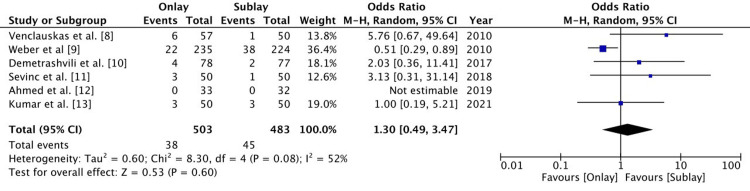
A forest plot of hernia recurrence The random effect method with the Mantel-Haenszel (M-H) test at a 95% confidence interval was used to assess the odds ratio. The forest plot on the right shows the pooled results (diamond) touching the vertical line, which indicates that the outcome of hernia recurrence is similar in both groups.

All six RCTs followed up on patients to assess hernia recurrence. Patients were followed up for a minimum of six months in all studies except for Kumar et al. [12] and Demetrashvili et al. [10], where the duration of follow-up was not recorded. Weber et al. [9] followed up with patients for five years. There were 81 cases of recurrence out of 986 patients. There was no statistically significant difference between the onlay and sublay groups (odds ratio (OR): 1.3 (0.49-3.47), 95% confidence interval (CI), p= 0.60). There was a significant level of heterogeneity (I2 = 52%) (Figure 3).

 

Postoperative complications

Five RCTs reported postoperative complications. The most common complications encountered were seroma, haematoma, and surgical site infection. Venclauskas et al. [8] also reported two cases of skin necrosis in the sublay group.

Seroma

**Figure 4:**
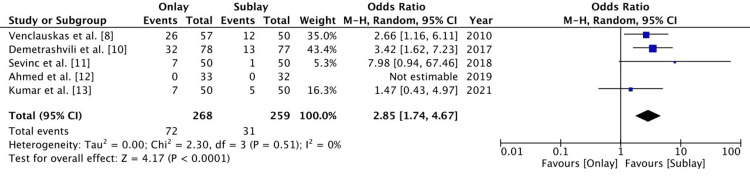
A forest plot for postoperative seroma The random effect method with the Mantel-Haenszel (M-H) test at a 95% confidence interval was used to assess the odds ratio. The forest plot on the right shows the pooled results (diamond) shifted to the right, which is in favour of the sublay group, indicating the risk of postoperative seroma is lower in the sublay group.

Five RCTs reported on postoperative seroma. There were 72 patients out of 268 patients in the onlay group and 31 patients out of 259 patients in the sublay group that developed a seroma. Seroma formation was significantly higher in the onlay group (OR 2.85 (1.74-4.67), 95% CI, p<0.0001). The level of heterogeneity was low (I2 = 0%) (Figure 4).

Surgical site infection

**Figure 5:**
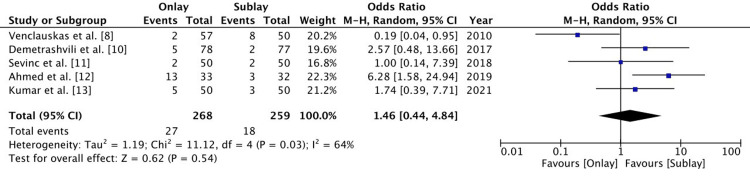
A forest plot for surgical site infection The random effect method with the Mantel-Haenszel (M-H) test at a 95% confidence interval was used to assess the odds ratio. The forest plot on the right shows the pooled results (diamond) touching the vertical line, which indicates that the risk of surgical site infection is the same in both groups.

There were five RCTs that reported on surgical site infections. There were 27 patients out of 268 patients in the onlay group and 31 patients out of 259 patients in the sublay group that developed a surgical site infection. There was no significant difference between the two groups (OR: 1.46 (0.44-4.84), 95% CI, p=0.54). The level of heterogeneity was significant (I2 = 64%) (Figure 5).

Haematoma

**Figure 6:**
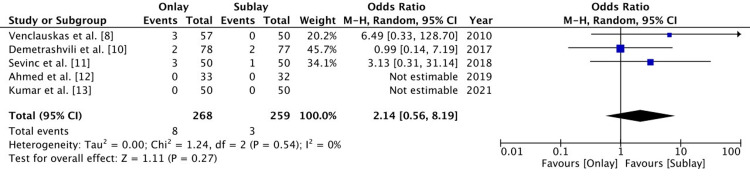
A forest plot for postoperative haematoma The random effect method with the Mantel-Haenszel (M-H) test at a 95% confidence interval was used to assess the odds ratio. The forest plot on the right shows the pooled results (diamond) touching the vertical line, which indicates that the outcome of postoperative haematoma is the same in both groups.

Five RCTs reported on postoperative haematoma. The haematoma was reported in eight patients out of 268 patients in the onlay group and in three patients out of 259 patients in the sublay group. There was no significant difference between the two groups, and the level of heterogeneity was low (OR 2.13 (0.56-8.19), 95% CI, p=0.27, I2 = 0%) (Figure 6).

Length of hospital stay

**Figure 7:**
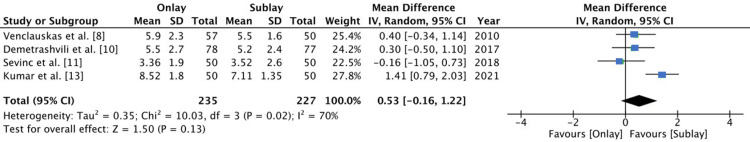
Forest plot of length of hospital stay The length of hospital stay was assessed using the mean difference, which was calculated using inverse variance (IV) with a random effect model at a 95% confidence interval. The forest plot on the right shows the pooled results (diamond) touching the vertical line, which indicates the length of hospital stay is similar for both groups.

Four RCTs reported the length of hospital stay. There was no significant difference between the two groups (mean difference (MD): 0.53 (-0.16 - 1.22), 95% CI, p=0.13) and there was significant heterogeneity (I2 = 70%) (Figure 7).

Discussion

IH repair is a common procedure performed worldwide and can prove to be technically challenging when these hernias are large in size. There have been numerous surgical techniques, ranging from open to laparoscopic repair. What varies between these approaches is the type of mesh being used and the plane in which the mesh is placed.

Onlay mesh repair is technically easier to perform as it involves dissection down to the anterior abdominal fascia, on which the mesh is secured. Since the mesh is placed more superficially, it increases the risk of seroma formation as well as the risk of surgical site infection [14-15]. Potential dead space that develops following dissection and raising of planes provides a space for seroma formation. Invariably, this space is greater in the onlay dissection, where the overlying layer is the subcutaneous fat and skin. In sublay dissection, a space is created between the rectus muscle and posterior rectus sheath, which is potentially a narrow space in comparison to onlay dissection. Our review found that the rate of seroma formation was significantly higher in the onlay group as compared to the sublay group. Perletti et al. [16] in their review found a significantly greater number of cases of seroma in the onlay group. Only two studies in the review mentioned the use of drains following surgery. The main purpose of placing a drain is to prevent any collection in the dead space so as to reduce seroma formation. There were 27 cases and 18 cases of surgical site infection reported in the onlay and sublay groups, respectively. There was no statistically significant difference between the two groups. Timmerman et al. [5] in their review did not find a significant difference between the two groups with respect to surgical site infection.

Sublay mesh placement involves dissection in the retrorectus plane so as to develop adequate space to place a mesh. This area will be more vascular, with greater chances of bleeding, as compared to an onlay dissection, which is relatively less vascular. Hence, the risk of haematoma formation is higher in sublay mesh repair [5]. In our review, haematomas were reported in 11 cases, but there were no significant differences between the two groups.

Factors affecting hospital stay postoperatively include early postoperative complications, postoperative pain, the duration of surgery, and the ASA score [17]. Only four RCTs [8,10,11,13] reported on the duration of hospital stay. None of the studies individually showed an advantage of one group over the other with respect to the length of hospital stay.

There are a number of factors that affect recurrence after IH repair. Common patient-related factors are age, high BMI, chronic illnesses, diabetes mellitus, and early physical exertion following surgery [18]. Technical factors that may contribute to recurrence include the size of the defect, type of procedure (i.e., suture versus mesh repair), wound infection, and type and plane of mesh placement [19-20]. Our review failed to show significant recurrence rates of one technique over the other, which had similar findings to a review conducted by Timmerman et al. [5].

Limitations

Although this review included six RCTs, the number of patients was relatively low, and the level of heterogeneity was moderate. Not all studies mention the type of mesh being used or if drains were kept intra-operatively. Only two studies comment on the mean size of the hernial defect, which is known to be a contributing factor to hernia recurrence; hence, this could not be used as a factor when assessing hernia recurrence.

## Conclusions

The current systematic review aims to compare onlay mesh repair with sublay mesh repair in the management of incisional hernias. The main outcomes assessed were hernia recurrence and postoperative complications. We cannot definitively conclude that one type of mesh repair is superior to the other due to the limitations mentioned in the study. However, we did find the rate of seroma formation was higher in the onlay group and the level of heterogeneity was low. We conclude that a large volume of standardised RCTs needs to be conducted in order to draw conclusive clinical recommendations.
